# Effect of microwave ablation treatment of hepatic malignancies on serum cytokine levels

**DOI:** 10.1186/s12885-020-07326-x

**Published:** 2020-08-26

**Authors:** Jing Zhao, Qiang Li, Merlin Muktiali, Bingjie Ren, Yingxi Hu, Dapeng Li, Zhi Li, Daoming Li, Yufeng Xie, Min Tao, Rongrui Liang

**Affiliations:** 1 Department of Radiation Oncology, the First Affiliated Hospital of Soochow University, Suzhou, China; 2 Department of Oncology, the First Affiliated Hospital of Soochow University, Suzhou, China; 3 Department of Lymphatic Hematologic Oncology, Jiangxi Cancer Hospital, Nanchang, China; 4 Department of Interventional Radiology, the First Affiliated Hospital of Soochow University, Suzhou, China; 5 Division of Neurosurgery, City of Hope Beckman Research Institute, Duarte, California USA

**Keywords:** Microwave ablation, Hepatic malignancy, Cytokines, IL-2, IL-6, Immunoregulation

## Abstract

**Background:**

Microwave ablation (MWA) is widely used to treat unresectable primary and secondary malignancies of the liver, and a limited number of studies indicate that ablation can cause not only necrosis at the in situ site but also an immunoreaction of the whole body. This study aimed to investigate the effects of MWA on cytokines in patients who underwent MWA for a hepatic malignancy.

**Methods:**

Patients admitted to the Oncology Department in the First Affiliated Hospital of Soochow University between June 2015 and February 2019 were selected. Peripheral blood was collected from patients with a hepatic malignancy treated with MWA. The levels of cytokines (IL-2, IFN-γ, TNF-α, IL-12 p40, IL-12 p70, IL-4, IL-6, IL-8, IL-10, and vascular endothelial growth factor (VEGF)) were detected with a Milliplex® MAP Kit. The comparison times were as follows: before ablation, 24 h after ablation, 15 days after ablation, and 30 days after ablation. Data were analyzed using a paired sample t-tests and Spearman’s correlation analysis.

**Results:**

A total of 43 patients with hepatic malignancies were assessed. There were significant differences in IL-2, IL-12 p40, IL-12 p70, IL-1β, IL-8, and TNF-α at 24 h after MWA. Significant increases (> 2-fold vs. before ablation) were observed in IL-2, IL-1β, IL-6, IL-8, IL-10, and TNF-α after MWA. Elevated IL-2 and IL-6 levels after ablation were positively correlated with energy output during the MWA procedure.

**Conclusions:**

WA treatment for hepatic malignancies can alter the serum levels of several cytokines such as IL-2 and IL-6.

## Background

 Primary and secondary malignancies of the liver have a substantial impact on morbidity and mortality worldwide. In China, hepatocellular carcinoma (HCC) has the second highest mortality rate of malignancies [1]. The treatment of primary and secondary hepatic malignancies via interventional imaging therapy is undertaken by investigators in the field of interventional radiology and possibly by a smaller group of practitioners known as interventional oncologists, whose major focus is cancer care via minimally invasive approaches [2, 3]. Recently, percutaneous ablation therapy has been widely accepted as a radical treatment method for HCC, and its five-year survival rate is similar to that of resection [4]. Microwave ablation (MWA) is widely used to treat unresectable HCC and recurrent HCC and has the advantages of minimal invasion, a good curative effect, and no side effects due to radiation or chemotherapy. Immune checkpoint inhibitors (ICIs), such as PD-1/PD-L1 and CTLA4 antibodies, have been widely applied in several cancers, and studies have indicated that ICI treatment could enhance the effect of ablation [5]. Evidence has indicated that hyperthermic destruction causes the release of a large population of heterogeneous tumor antigens, and inflammatory cytokines may play crucial roles in this process [6]. Cytokines are mediators that regulate a broad range of processes involved in the pathogenesis of cancer. Several cytokines, which can arise from either tumor cells or immunocytes [7], such as tumor necrosis factor (TNF)-α, interleukin (IL)-1β, IL-6, IL-8, IL-10, and vascular endothelial growth factor (VEGF), have been linked with cancers and can either promote or inhibit tumor development. The serum levels of cytokines differ during cancer development. Although cytokines have been found to be altered after anticancer treatment, such as chemotherapy and radiotherapy [8, 9], few investigations have focused on cytokines before and after MWA. It is still unknown whether the above cytokines changed before and/or after MWA in patients with hepatic malignancies. In this study, we investigated the effects of MWA on the serum levels of cytokines in patients with hepatic malignancies.

## Methods

### Patients and samples

The patient population examined in this study was derived from the First Affiliated Hospital of Soochow University. Patients were admitted to the Oncology Department between June 2015 and February 2019. The total number of patients was 43, with 37 liver metastases and 6 primary liver cancers. The inclusion criterion was a tumor located at a hepatic site (either primary or metastases). All patients with metastatic hepatic malignances should be given systematic treatments (chemotherapy or target therapy) and get at least stable disease (SD) or partial response (PR) for more than 45 days. Informed consent for blood draw and the relevant therapy was obtained from all patients. The protocol was approved by the Human Ethics Committee of the First Affiliated Hospital of Soochow University and was conducted in accordance with the Declaration of Helsinki. All written informed consent, was obtained from all participants and clearly stated. Whole blood (4 mL) was drawn into EDTA anticoagulant tubes on days − 3 to 0 before and 24 h, 15 days, and 30 days after ablation, mostly on the last day of the course, for cytometry and cytokine analyses.

### Ablation procedure

The ablation procedure used in this research was MWA. The puncture site and pathway were determined under the guidance of a computed tomography (CT) scan. Local infiltration anesthesia was achieved by using 0.5% lidocaine. The placement of microwave ablation probes was guided by a CT scan or ultrasonic device, and all probes were placed at the maximum diameter layer. Double probes were employed when the maximum diameter of the tumor was up to 3 cm. The power and time of ablation were designed for each patient in the range of 40 ~ 70 W and 5 ~ 20 min, respectively, based on the size, number, and position of the tumor. The boundaries of ablation zones were designed as extended 1 cm upon the tumor site.

### Cytokine detection

A Milliplex MAP Kit with 10 human cytokine/chemokine panels that measured IFN-γ, IL-2, IL-6, IL-8, IL-10, IL-12 p40, IL-12 p70, IL-1β, TNFα, and VEGF was utilized according to the manufacturer’s instructions. Briefly, chemically dyed antibody-bound beads were mixed with standard or sample, incubated overnight at 4 °C, washed, and then incubated with a biotinylated detection antibody. After the beads were washed, they were incubated with a streptavidin phycoerythrin complex, and the mean fluorescent intensities were quantified on a Luminex 200 analyzer (Luminex Corporation). All samples were measured in duplicate. Standard curves of known concentrations of recombinant human cytokines/chemokines were used to convert fluorescence units to cytokine concentration units (pg/mL). The minimum detectable concentrations were as follows: IFN-γ: 2.6 pg/mL, IL-2: 2.77 pg/mL, IL-12 p40: 3.94 pg/mL, IL-12 p70: 2.84 pg/mL, IL-1β: 2.99 pg/mL, IL-6: 2.79 pg/mL, IL-8: 2.92 pg/mL, IL-10: 2.42 pg/mL, TNF-α: 3.3 pg/mL, and VEGF: 1.5 pg/mL. All results below the minimum concentrations were processed as the minimum concentrations.

### Statistical analysis

IBM SPSS Statistics 20.0 software was used for the statistical analysis, along with GraphPad Prism 8 for figure creations. Normally distributed numerical data are expressed as the mean ± standard deviation, and nonnormally distributed numerical data are expressed as the median and 95% confidence interval (95% CI). Cytokines at different times were compared using a one-tailed paired t-test. Spearman’s correlation analysis was executed to determine the correlation between clinical indexes and cytokine levels. *p* < 0.05 indicates a significant difference.

## Results

### Clinical characteristics of the enrolled patients

As shown in Table 1, a total of 43 patients with tumors located on the liver (37 liver metastases, 6 primary liver cancers) were analyzed. The patients’ cytokine levels were compared according to time: before treatment, 24 h after treatment, 15 days after treatment, and 30 days after treatment.

**Table 1:** Clinical characteristics of the patients enrolled. (*n* = 43)

Characteristic	
**Sex**	
male	28
female	15
**Age**	62.81 ± 8.14
**Pathogenesis**	
primary	6
secondary	37
**Primary site (For metastatic hepatic malignances)**	
Colon & rectal	18
Pancreas	7
Stomache	3
Breast	3
Others	9
**Maximum tumor length (mm)**	33.58 ± 13.29
**Ablation probe used**	
1	31
2	12
**Ablation time (min)**	27.09 ± 14.36
**Average power per probe (W)**	51.98 ± 5.13
**Average energy (time 1 × power 1 + time 2 × power 2)**^**▼**^	1426 ± 806

▼, Time 1/2 and power 1/2 indicate the time and power, respectively, of different probes used during the operation

### IFN-γ, IL-12 p40, and IL-12 p70 were slightly increased after MWA treatment

**Fig. 1:**
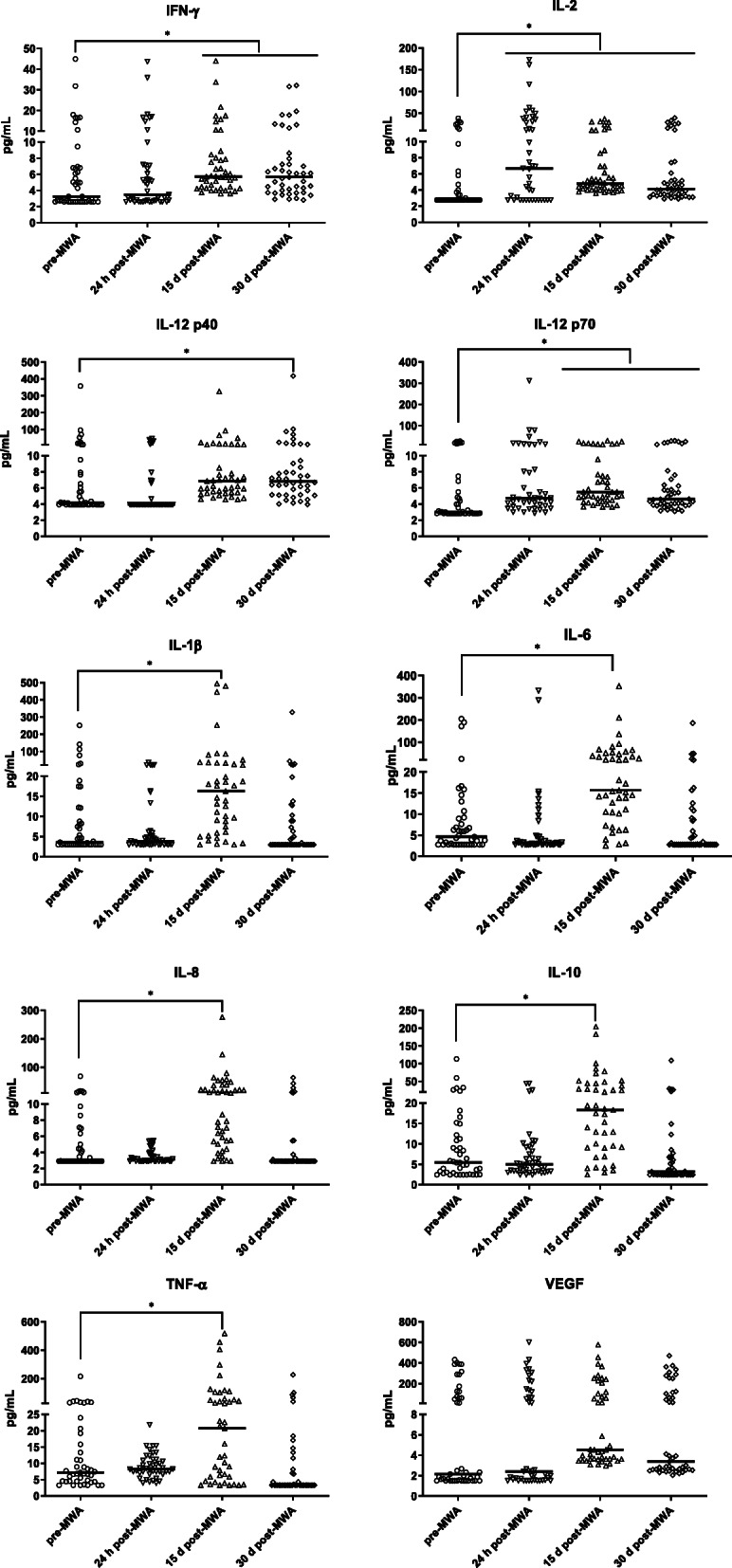
Levels of cytokines before and after MWA treatment. Slightly increased IFN-γ, IL-12 p40, and IL-12 p70 levels after MWA treatment. Over 2-fold enhancement of IL-2 24 h post-MWA and of IL-1β, IL-6, IL-8, IL-10 and TNF-α 15 d post-MWA. *, *p* < 0.05

As shown in Table 2 and Fig. 1, the median level of IFN-γ before the MWA treatment was 3.25 pg/mL (95% CI 2.72–6.12 pg/mL); at 15 days and 30 days after the MWA treatment, there was a slight increase compared to that pre-MWA, with median levels of 5.71 pg/mL (95% CI 5.15–7.51 pg/mL) and 5.65 pg/mL (95% CI 4.47–6.71 pg/mL), respectively. The median level of IL-12 p40 before the MWA treatment was 4.16 pg/mL (95% CI 3.94–5.56 pg/mL). There was a slight increase to 6.81 pg/mL (95% CI 6.17–7.90 pg/mL) 30 days post-MWA. The median IL-12 p70 level before the MWA treatment was 3.00 pg/mL (95% CI 2.84–4.01 pg/mL) and increased to 5.49 pg/mL (95% CI 4.90–6.79 pg/mL) 15 days after the MWA treatment and to 4.61 pg/mL (95% CI 4.07–5.75 pg/mL) 30 days post-MWA. No significant alteration in the VEGF median level was detected after the MWA treatment.

**Table 2:** Median levels of cytokines before and after MWA

Cytokine	pre-MWA (pg/mL)	24 h post-MWA (pg/mL)	15 days post-MWA (pg/mL)	30 days post-MWA (pg/mL)
IFN-γ	3.250 (95% CI 2.72–6.12)	3.47 (95% CI 2.80–5.39)	5.71 (95% CI 5.15–7.51) ^*^	5.65 (95% CI 4.47–6.71) ^*^
IL-2	2.77 (95% CI 2.77–3.38)	6.69 (95% CI 3.31–11.56)^*, ▼^	4.82 (95% CI 4.41–5.53) ^*^	4.14 (95% CI 3.51–4.89) ^*^
IL-12 p40	4.16 (95% CI 3.94–5.56)	3.94 (95% CI 3.94–3.94)	6.86 (95% CI 5.90–7.63)	6.81 (95% CI 6.17–7.90) ^*^
IL-12 p70	3.00 (95% CI 2.84–4.01)	4.72 (95% CI 4.17–6.01)	5.49 (95% CI 4.90–6.79) ^*^	4.61 (95% CI 4.07–5.75) ^*^
IL-1β	3.58 (95% CI 2.99–7.46)	3.80 (95% CI 3.22–4.55)	16.30 (95% CI 9.67–19.79) ^*,▼^	2.99 (95% CI 2.99–5.00)
IL-6	4.63 (95% CI 3.00–6.72)	3.22 (95% CI 2.86–3.65)	15.68 (95% CI 13.90–26.99) ^*,▼^	2.79 (95% CI 2.79–4.90)
IL-8	2.92 (95% CI 2.92–4.19)	3.15 (95% CI 2.92–3.45)	10.07 (95% CI 6.38–19.91) ^*,▼^	2.92 (95% CI 2.92–2.92)
IL-10	5.42 (95% CI 3.66–9.01)	4.95 (95% CI 3.50–6.33)	18.32 (95% CI 12.71–26.22) ^*,▼^	3.17 (95% CI 2.42–5.46)
TNF-α	7.19 (95% CI 5.53–10.97)	8.29 (95% CI 7.49–9.81)	20.77 (95% CI 7.87–37.85) ^*,▼^	3.30 (95% CI 3.30–4.28)
VEGF	2.15 (95% CI 1.66–48.85)	2.39 (95% CI 1.77–50.28)	4.53 (95% CI 3.67–53.47)	3.39 (95% CI 2.66–44.77)

*, *p* < 0.05 vs. pre-MWA; ▼, > 2-fold vs. pre-MWA

### IL-2, IL-1β, IL-6, IL-8 and IL-10 were elevated over 2-fold after the MWA treatment

**Fig. 2:**
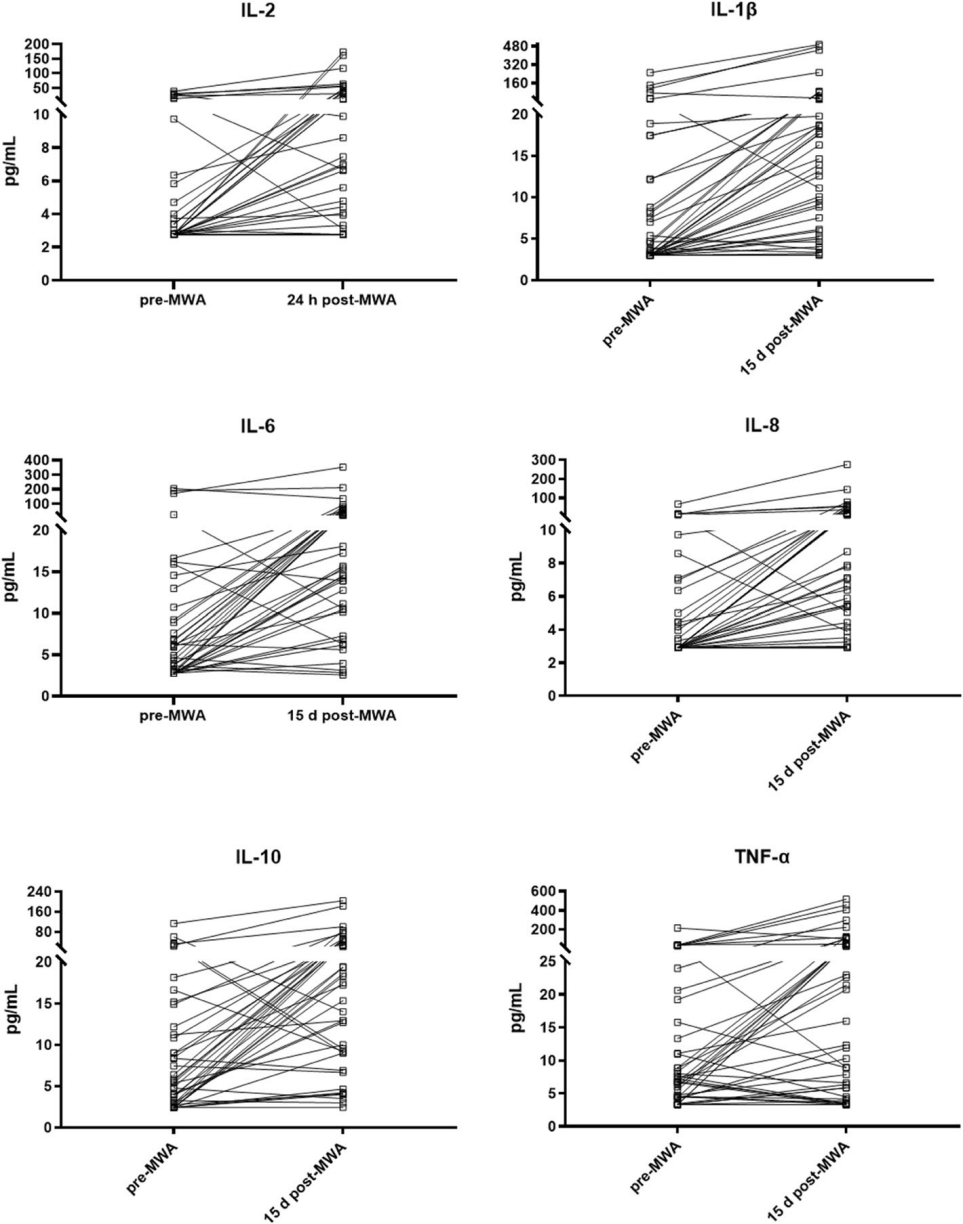
Trends in cytokines significantly altered after MWA treatment. The levels of IL-2 at 24 h post-MWA, IL-1β at 15 d post-MWA, IL-6 at 15 d post-MWA, IL-8 at 15 d post-MWA and IL-10 at 15 d post-MWA were elevated over 2-fold compared to the levels pre-MWA

As shown in Table 2, Fig. 1 and Fig. 2, the median level of IL-2 before the MWA treatment was 2.77 pg/mL (95% CI 2.77–3.38 pg/mL). There was a significant increase at 24 h post-MWA, with a median level of 6.69 pg/mL (95% CI 3.31–11.56 pg/mL). The median level of IL-1β before the MWA treatment was 3.58 pg/mL (95% CI 2.99–7.36 pg/mL), and a significant increase was noted 15 days after the MWA treatment (16.30 pg/mL) (95% CI 9.67–19.79 pg/mL). The median level of IL-6 before the MWA treatment was 4.63 pg/mL (95% CI 3.00–6.72 pg/mL) and significantly increased 15 days after the MWA treatment (15.68 pg/mL) (95% CI 13.9–26.99 pg/mL). The median level of IL-8 before the MWA treatment was 2.92 pg/mL (95% CI 2.92–4.19 pg/mL) and increased significantly to 10.07 pg/mL (95% CI 6.38–19.91 pg/mL) 15 days after the MWA treatment. The median level of IL-10 before the MWA treatment was 5.42 pg/mL (95% CI 3.66–9.01 pg/mL) and increased significantly 15 days after the MWA treatment (18.32 pg/mL) (95% CI 12.71–26.22 pg/mL). The median level of TNF-α before the MWA treatment was 7.19 pg/mL (95% CI 5.53–10.97 pg/mL) and increased significantly to 20.77 pg/mL (95% CI 7.87–37.85 pg/mL) 15 days after the MWA treatment.

### Elevated IL-2 and IL-6 levels after ablation were positively correlated with energy output during MWA

**Fig. 3:**
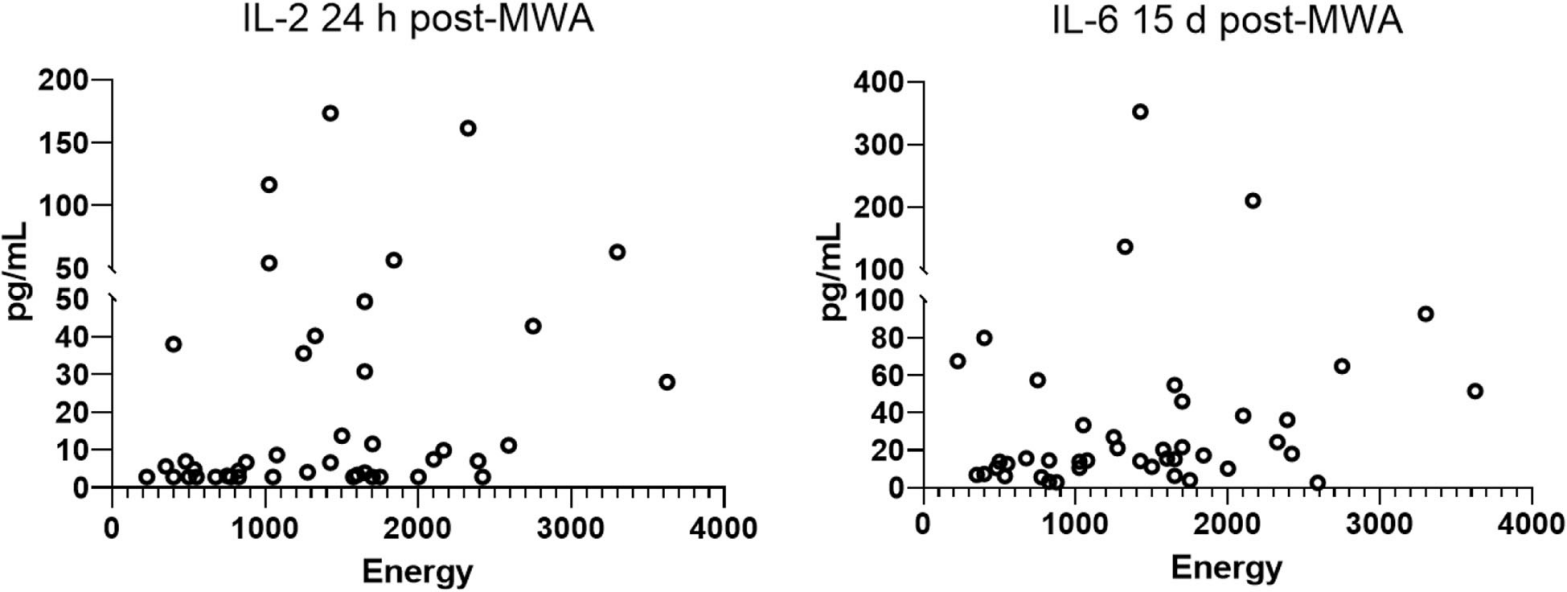
Correlation between the ablation energy and the serum levels of IL-2 and IL-6. The serum levels of IL-2 at 24 h post-MWA and IL-6 at 15 d post-MWA were positively correlated with energy output during the MWA procedure

To further evaluate the relationship between the increased cytokine levels and MWA treatment, we employed the concept of “energy” (time 1 × power 1 + time 2 × power 2, time 1/2 and power 1/2 indicated the time and power of different probes used in the operation) to reflect total hyperthermic damage to hepatic tissues during the MWA procedure. As shown in Table 3 and Fig. 3, the IL-2 levels at 24 h post-MWA and the IL-6 levels at 15 days post-MWA illustrated significant correlations with energy; the relative indexes were 0.35 and 0.29, respectively.

**Table 3:** Correlation between the ablation energy and significantly elevated cytokines

	Energy vs. IL-2 24 h post-MWA	Energy vs. IL-1β 15 d post-MWA	Energy vs. IL-6 15 d post-MWA	Energy vs. IL-8 15 d post-MWA	Energy vs. IL-10 15 d post-MWA	Energy vs. TNF-α 15 d post-MWA
Spearman’s r	0.3501	−0.06957	0.2911	0.1986	0.1205	0.04439
*P* value (one-tailed)	0.0107*	0.3288	0.0291*	0.1009	0.2208	0.3887

*, *p* < 0.05

## Discussion

As technology continues to develop, other types of local therapy, such as radiotherapy, chemical ablation and hyperthermal ablation, for primary and metastatic liver cancer are increasingly being used. MWA for liver malignances is reserved for patients who cannot undergo surgical removal or for whom other treatments have failed [10]. A consensus guideline was recently developed to address indications for MWA in these patients. Thermal ablation is a process that heats the target tissue to a temperature that causes immediate coagulative necrosis (usually over 100 °C). Terminal treatment requires that a necrotic area surrounds the target site with an additional 5–10-mm margins [11]. However, in the liver, high tissue perfusion and large blood vessels can cause a “heat sink effect” around the ablation zone, making it difficult to achieve terminal ablation [12]. The heat sink effect can lead to sublethal temperatures and the retention of malignant cells, thereby increasing the likelihood of local tumor progression (LTP) [13]; however, an incompletely ablated zone containing immune cells and cancer cells, as well as functional vessels, could establish a serious inflammatory site that may provide tumor-specific antigens, cytokines, and activated immune cells.

In our study, significant increases in the secretion of chemokines (IL-8), proinflammatory cytokines (IL-1β, IL-12, IFN-γ and TNF-α) and anti-inflammatory cytokines (IL-10) were observed after MWA. IL-8 is mainly produced by macrophages, the classical biological activity of IL-8 is to attract and activate neutrophils, which can lead to a local inflammatory response. However, recent studies have indicated that IL-8, both macrophage- and cancer cell-derived, can recruit Myeloid-derived suppressor cells (MDSCs) into the tumor microenvironment, eventually inhibiting antitumor immunity and promoting cancer progression [14, 15]. IL-1β is mainly produced by macrophages., B cells and NK cells, could produce IL-1β under certain circumstances. Generally, cells can only synthesize and secrete IL-1β after being stimulated by foreign antigens or mitogens. IL-1β could promote the Th1 response, promoting the activation of Dendritic cells (DCs) and Cytotoxic T lymphocytes (CTLs). IL-12 is mainly produced by B cells and macrophages. Human IL-12 is a heterodimer with two subunits: p40 (40 kD) and p35 (35 kD), which are inactivated in isolated form. In general, IL-12 functions as a combination of two subunits (IL-12 p70), while p40 alone possesses partial functions of IL-12 p70. It’s mentionable that IL-12 p40 and p35 are not expressed in equal proportions, so the amounts of IL-12 p40 and IL-12 p70 are different in one cell. IL-12 can stimulate the proliferation of activated T cells and promote the differentiation of Th0 cells into Th1 cells. Moreover, IL-12 could induce the cytotoxic activity of CTLs and NK cells and promote the secretion of several cytokines, such as IFN-γ [16] and TNF-α [17]. Previous research indicated that TNF-α may play a crucial role in MWA in combination with immunotherapy [18]. Notably, our data illustrated that the IL-12 results were consistent with those of IFN-γ after the ablation operation but not with those of TNF-α. This result indicated that upregulation of IFN-γ may be a major effect of the IL-12 increase after MWA. On the other hand, IL-10, as an anti-inflammatory and immunosuppressive cytokine, was evaluated after MWA. IL-10 is a multicellular-derived, multifunctional cytokine that regulates cell growth and differentiation and could participate in inflammatory and immune responses. IL-10 was reported to increase after thermal ablation in the literature [19, 20]. Strategies to inhibit IL-10-induced immunosuppression after thermal ablation treatment would be of interest.

Ablation therapy can mediate antitumor immunity, as tumor tissue necrosis caused by ablation may release various antigens that eventually form a kind of “in situ vaccination” [21]. Moreover, ablative therapy can not only directly kill cancer cells in situ but also regulate immune cells and promote the immune function of patients with liver cancer [22, 23]. Many immunoregulatory cytokines were released or expressed after thermal ablation. Notably, the cytokines released after thermal ablation can regulate the positive and negative aspects of the cancer immune cycle. Previously, researchers demonstrated that proinflammatory cytokines such as IL-1, IL-6, IL-8, IL-18, and TNF-α were increased several hours or days after thermal ablation [20, 24, 25]. To our knowledge, terminal tumor thermal ablation may not only cause local heat injury in tissues surrounding the tumor site but also induce a systemic reaction [26]. This systemic reaction would be caused by different mechanisms. First, interventional operation may result in trauma to the liver although this procedure is very minimally invasive, the healing process may cause alteration of some cytokines. Second, heat injury could cause acute thermal necrosis in liver and tumor tissues, and release of necrotic tissue fragments into blood could cause immunological reactions, including nonspecific and specific reactions. Generally, cytokines affected by wound healing and nonspecific immunological reactions do not last longer than those affected by specific immunological reactions. Ablation treatment-induced specific immunological reactions are more complicated and could affect more immunocytes [27, 28], which would make this process last longer than other reactions. These explanations may be the reason why the cytokine changes lasted different durations. Moreover, cytokines affected by the second manner would be positively correlated with the ablation scale, which is why we employed the “energy” index. In our ablation operation design, to receive a terminal ablation, larger tumor would cost higher energy, including higher power and longer duration time. Terminal tumor thermal ablation would release tumor-related neoantigen to blood circulation, eventually induce a systemic reaction. This reaction is dependent on the scale of thermal injury and the local immunological microenvironment of the tumor. Our findings indicated that IL-2 and IL-6 were significantly altered after the ablation procedure and positively correlated with MWA energy. IL-2 is commonly derived from activated T cells, primarily Th1 cells. IL-2 can stimulate T cells to proliferate and differentiate, activate natural killer (NK) cells and macrophages, and enhance the functions of cytotoxic T lymphocytes (CTLs) [29]. Our data illustrated that IL-2 is significantly increased at 24 h after MWA, indicating that IL-2 may induce a nonspecific immune response after MWA. But IL-12 decreased after 24 h post-MWA in our study, suggesting that the IL-2-induced immune response may not be long lasting. Mentionable, many cytokines detected (IL-8, IL-1β, IL-12) were mainly derived from macrophage, which was a widely distributed antigen presenting cell. This result support the theory that MWA could release fragment of cancer cells into blood as neoantigen, macrophages could response to this proceed and cause a systemic immunoreaction. Additional, cytokines alteration such as IL-6 after ablation may be no organ-specific in liver. Evidences indicate that increase of IL-6 was not only occurred in liver ablation, researches focus on lung cancer, including primary lung cancer and pulmonary metastases demonstrated that serum IL-8, IL-1β, IL-6, IL-10, IL-12 and TNF-α were significantly raised after radiofrequency thermal ablation [25]. Moreover, Joseph found that image-guided thermal ablation of tumors located in lung, liver or soft tissues increases plasma levels of IL-6 and IL-10 [20]. Another question remain unveiled was if our result was “cancer-specific”. We checked literature about cytokine modulation after thermal ablation in benign diseases and only got limit evidences based on benign thyroid nodules [30] and adenomyosis [31]. According to these literature, IL-6 levels did not show any significant difference after treatment compared with pretreatment values, indicating that elevation of IL-6 may be caused by tumour antigen released by ablation treatment. However, the ablation energy used in thyroid nodules was much lower than liver and lung, which would lead to a false negative in cytokine detection. To the research about adenomyosis, on the other hand, experiment design was determined to follow-up the IL-6 at 6 months after HIFU ablation. As our data demonstrated, mostly cytokines were return to pre-MWA level after 1 month, detection after 6 months may miss the modulation of IL-6. Overall, few evidences support that some of the cytokines were altered in a “cancer-specific” manner while no solid results could confirm that. Further animal experiments were required to make a clarified data and answer this question.

In recent years, ablation-induced systemic effects, such as the tumor-associated immune response, have attracted increased attention [32]. de Baere T first reported two cases of spontaneous regression of multiple pulmonary metastases occurring after radiofrequency ablation of a single lung metastasis [33]. Although growing evidence suggests that thermal ablation can induce spontaneous regression of the so-called “abscopal effect” on distant tumors, the incidence rate of such an effect is rare, probably due to uncontested immunological activation caused by one ablation treatment and the lack of immune-amplification management. In 2004, it was described that in situ tumor destruction can provide a useful antigen source for the induction of antitumor immunity [26]; however, clinical studies could not sufficiently utilize such an effect until the development of immune checkpoint inhibitors (ICIs) [34, 35]. ICIs such as PD-1/PD-L1 and CTLA4 antibodies, are widely applied in several cancers, and studies have indicated that ICI treatment could enhance the effect of ablation [5]. Evidence indicates that hyperthermic destruction causes the release of a large population of heterogeneous tumor antigens, and inflammatory cytokines may play crucial roles in this process [6]. However, opposite evidence indicated that incomplete radiofrequency ablation could induce inflammation which may accelerates tumor progression and hinders PD-1 immunotherapy [36], suggesting that ablation treatment may promote tumor progression. Our data demonstrated that IL-6 was significantly increased after MWA treatment, IL-6 is derived from monocytes, macrophages, DCs, Th2 cells and sometimes cancer cells, and it plays a key role in T cell proliferation and survival [37]. The role of IL-6 appears to be rather complex, Korn classified IL-6 as “differentiation factor” which could involve in differentiation of Th17 cells [38]. However, IL-6 does not direct the commitment to the Th1 or Th2 cell lineage, but controls the proliferation and survival of immunocytes cooperating with other cytokines such as TGF-β, TNF or IL-21 [39]. For instance, IL-6 activated STAT3 pathway in naive CD4^+^ T cells in the presence of the morphogen TGF-b promotes the population expansion of Th17 cells [40, 41]. Recent evidence indicates that IL-6 plays an indispensable role in T cell infiltration to the tumor site, which could benefit immunomodulatory therapy [42]. In contrast, IL-6 can increase MDSCs [43], inhibit the development and maturation of dendritic cells (DCs) [44], and inhibit the polarization of Th1 cells [45], eventually resulting in negative immunomodulatory effects. According to Muneeb Ahmed’s work, the adjuvant uses of a nanoparticle small interfering RNA (siRNA) can be successfully used to target the IL-6-mediated locoregional and systemic effects of thermal ablation. IL-6 knockout via a nanoparticle anti-IL-6 siRNA in mice could decrease the local VEGF level at the ablation site [46]. Therefore, how to utilize the positive effect of IL-6 while avoiding the negative effect after MWA needs further investigation. Preclinical research indicated that IL-6 and PD-L1 blockade combination therapy reduced tumor progression in animal models [47, 48]. Thus, an anti-IL-6 strategy after ablation should be considered when combined with ICI therapy. Previous studies and ours have demonstrated that most cytokine levels returned to pretreatment levels 30 days after ablation. This result suggests that 24 h to 15 days after ablation may be optimal timing for additional immunomodulatory therapy.

## Conclusions

Our results reported here support the evidence for terminal tumor thermal ablation could cause heat injury to tissues surrounding the tumor site and release neoantigen to blood circulation, eventually induce a systemic reaction. This reaction could lead to a detectable alteration of cytokine levels. Further investigation is required to reveal whether the cytokines altered by MWA treatment could affect cancer progression, whether positive or negative.

## Data Availability

The datasets used and/or analysed during the current study are available from the corresponding author on reasonable request.

## Abbreviations

MWA: Microwave ablation
HCC: Hepatocellular carcinoma
ICIs: Immune checkpoint inhibitors
TNF: Tumor necrosis factor
IL: Interleukin
VEGF: Vascular endothelial growth factor
SD: Stable disease
PR: Partial response
CT: Computed tomography
CI: Confidence interval
LTP: Likelihood of local tumor progression
MDSCs: Myeloid-derived suppressor cells
CTLs: Cytotoxic T lymphocytes
NK: Natural killer
siRNA: Small interfering RNA
